# Developing a Shared Patient-Centered, Web-Based Medication Platform for Type 2 Diabetes Patients and Their Health Care Providers: Qualitative Study on User Requirements

**DOI:** 10.2196/jmir.8666

**Published:** 2018-03-27

**Authors:** Gerda Bernhard, Cornelia Mahler, Hanna Marita Seidling, Marion Stützle, Dominik Ose, Ines Baudendistel, Michel Wensing, Joachim Szecsenyi

**Affiliations:** ^1^ Department of General Practice and Health Services Research Heidelberg University Hospital Heidelberg Germany; ^2^ Cooperation Unit Clinical Pharmacy Heidelberg University Hospital Heidelberg Germany; ^3^ Department of Clinical Pharmacology and Pharmacoepidemiology Heidelberg University Hospital Heidelberg Germany; ^4^ Division of Cancer Population Sciences Department of Population Health Sciences University of Utah Salt Lake City, UT United States

**Keywords:** medication, self-management, patient portal, patient participation, type 2 diabetes mellitus, focus groups, primary care

## Abstract

**Background:**

Information technology tools such as shared patient-centered, Web-based medication platforms hold promise to support safe medication use by strengthening patient participation, enhancing patients’ knowledge, helping patients to improve self-management of their medications, and improving communication on medications among patients and health care professionals (HCPs). However, the uptake of such platforms remains a challenge also due to inadequate user involvement in the development process. Employing a user-centered design (UCD) approach is therefore critical to ensure that user’ adoption is optimal.

**Objective:**

The purpose of this study was to identify what patients with type 2 diabetes mellitus (T2DM) and their HCPs regard necessary requirements in terms of functionalities and usability of a shared patient-centered, Web-based medication platform for patients with T2DM.

**Methods:**

This qualitative study included focus groups with purposeful samples of patients with T2DM (n=25), general practitioners (n=13), and health care assistants (n=10) recruited from regional health care settings in southwestern Germany. In total, 8 semistructured focus groups were conducted. Sessions were audio- and video-recorded, transcribed verbatim, and subjected to a computer-aided qualitative content analysis.

**Results:**

Appropriate security and access methods, supported data entry, printing, and sending information electronically, and tracking medication history were perceived as the essential functionalities. Although patients wanted automatic interaction checks and safety alerts, HCPs on the contrary were concerned that unspecific alerts confuse patients and lead to nonadherence. Furthermore, HCPs were opposed to patients’ ability to withhold or restrict access to information in the platform. To optimize usability, there was consensus among participants to display information in a structured, chronological format, to provide information in lay language, to use visual aids and customize information content, and align the platform to users’ workflow.

**Conclusions:**

By employing a UCD, this study provides insight into the desired functionalities and usability of patients and HCPs regarding a shared patient-centered, Web-based medication platform, thus increasing the likelihood to achieve a functional and useful system. Substantial and ongoing engagement by all intended user groups is necessary to reconcile differences in requirements of patients and HCPs, especially regarding medication safety alerts and access control. Moreover, effective training of patients and HCPs on medication self-management (support) and optimal use of the tool will be a prerequisite to unfold the platform’s full potential.

## Introduction

### Medication Self-Management in Type 2 Diabetes

The prevalence of type 2 diabetes mellitus (T2DM) is steadily growing and represents a challenge for health care systems worldwide [1]. The complex nature of T2DM management requires ongoing efforts by the patient in collaboration with health care professionals (HCPs) and informal support networks. Compared with other chronic conditions, T2DM management requires extensive self-management behaviors of patients, such as adhering to often complex medication regimens, lifestyle modifications (eg, diet, physical activity), recognizing and responding to symptoms, and managing acute episodes [2]. Patient education and promoting patients’ self-management has long been recognized as an important strategy in diabetes management [3].

Medication self-management, defined as the various tasks patients must undertake to effectively manage their therapeutic regimen and sustain safe medication use over the long term [4], is a critical skill for patients with T2DM. Considering this definition, taking medications is a complex, multistep task and requires patients filling the prescription, understanding the medication regimen, organizing and correctly taking the medication, monitoring the intake, and then sustaining medication use over time [4]. Research has demonstrated that patients with T2DM often have an inadequate understanding on how to safely take their medications and concerns regarding the appropriateness and safety of their regimen [5,6]. Inadequate understanding can lead to improper use, adverse drug events (ADEs), and suboptimal adherence [7-9]. In fact, suboptimal medication adherence is common among patients with T2DM [10] and is associated with poor health outcomes [11].

### Health Information Technology

Health information technology (HIT) that is patient-centered [12], such as Web-based personal health records, creates new opportunities to facilitate patients’ diabetes and medication self-management and enhances patient outcomes [13-15]. The use of these technologies can provide patients access to personal medication information and essential resources to facilitate informed decision making, promote communication between patients and HCPs, and enhance patient engagement and self-management [14,16], particularly in patients with chronic conditions such as T2DM [16,17]. However, despite the potential benefits of HIT, introducing new technology to health care has proven difficult [18] and adoption rates are often low [19]. Factors identified in the literature that inhibit successful HIT implementation include inadequate funding, lack of IT infrastructure, poor leadership, unrealistic timeline, and inadequate end-user engagement and input [18,20]. Hence, research emphasized the need to directly involve intended users in requirement specification, design, and testing to ensure that HIT matches users’ cognitive abilities and needs, and support self-managed care [19].

### User Requirements Elicitation

Utilizing a user-centered design (UCD) [21] will enhance closer user participation throughout the entire development process and result in better tailoring to user requirements and needs [22,23]. Likewise, the FITT framework (Fit between Individuals, Task, and Technology) highlighted that successful adoption of a technology depends on the fit between the attributes of the individual user, the task, and the technology [24].

Today, a vast number of mobile apps exist to help manage outpatient medication use for diverse medical conditions. A systematic review [25] found that most apps provided medication reminders and half enabled creating a medication history, list, or log, whereas only few helped patients to organize their regimen and check for drug interactions. Overall, the quality, content, and functionality varied greatly. Common user criticism revealed technical malfunctions, poor compatibility with complex or varying regimes, and absence of desired features. The review concluded that further research is necessary to improve the design, content, and features from a patient perspective [25]. To date, only a few studies [14,26,27] have reported user requirements regarding the design and development of patient-centered HIT to support T2DM patients in self-managing their medications [28]. Requirements reported in these studies included reliable information on medication side effects and interactions, electronic messaging, selectively disclosing information, refill reminder functionality, and a user-friendly format [14,26,27]. Although these studies provide valuable information on user requirements, they have not elicited the views of both user groups: patients and HCPs. In Germany, diabetes care is largely provided in primary care with general practitioners (GPs) and health care assistants (HCAs) playing a key role in coordinating care, pharmaceutical treatment, and patient education. Thus, involving patients as well as GPs and HCAs as collaborative partners will help to identify what functionalities users require to accomplish the medication management tasks. Moreover, previous research has highlighted that HCPs endorsement of HIT is pivotal to increase patients’ participation and sustain use over time [29,30]. E-medication is a primary objective in Germany’s eHealth strategy; however, the development of a national e-medication infrastructure is slow and cumbersome [31].

Therefore, this study employed a UCD to identify necessary requirements in terms of functionalities and usability of a shared patient-centered, Web-based medication platform from the perspective of patients with T2DM and their HCPs (GPs and HCAs). Patients should be able to access, share, manage, and maintain personal medication-related information through this platform, with the intention of enhancing patients’ knowledge and strengthening patients’ active participation, thus helping them to better self-manage their medications and support safe medication use.

## Methods

### Research Design

A qualitative study based on focus groups was employed to explore the views of patients with T2DM and HCPs on their requirements of a shared patient-centered, Web-based medication platform [32,33]. Focus groups provide the opportunity for a group of people to explore and clarify their perspectives than would arise in individual interviews, facilitated by social interaction. This qualitative method is thus particularly useful for exploring not only what people think but also how they think and why they think that way [32]. The study was conducted as part of a larger German research project called INFOPAT (Information Technologies for Patient-Centered Health Care, 2012-2016) [34] studying the needs of chronically ill patients and their HCPs to develop tailored information technologies (ie, Web-based medication platform) and a medication communication intervention to facilitate medication self-management, improve medication safety, and patient-provider communication. Recruitment and study procedures have also been described in detail previously [35,36].

### Study Participants and Recruitment

Between April and July 2013, participants were purposefully recruited from the Rhine-Neckar region in southwestern Germany. The purposive sampling strategy aimed to achieve variation in patient characteristics, including education, duration of diabetes, and medication regimen to elicit a broad range of experiences and requirements. German or Turkish-speaking adults (largest ethnic minority in Germany) with a T2DM diagnosis who were self-administering prescribed diabetes medications (oral hypoglycemic agents [OHA] only or insulin only or OHA and insulin) were approached personally through 3 channels: local self-help groups, GP practices, and during routine appointments at the Heidelberg University Hospital. There were no restrictions on age, nor was computer or internet experience a prerequisite to take part in the study.

GPs and HCAs with diabetes expertise, and experienced in caring for T2DM patients, were recruited by a letter through a list of cooperating academic teaching and research practices from different geographic locations. The sampling approach aimed to ensure diversity in terms of practice size, urban/rural location, and computerization in practice. All participants gave informed written consent before study enrollment. Ethical approval was granted by the Ethics Committee of the Medical Faculty of Heidelberg University (no S-673/2012). Participants received a compensation of 50 €.

### Focus Groups

From May until July 2013, 8 focus groups with a total of 48 individuals, including 25 patients with T2DM, 13 GPs, and 10 HCAs, were conducted. Focus groups were conducted with 6-8 participants per group. Of these 8 groups, 4 groups included only T2DM patients, 3 groups included GPs and HCAs, and 1 group included only GPs. Each session was facilitated in German by an experienced moderator and comoderator (authors GB, CM, or DO) with the assistance of a trained note taker. The meeting with the group of patients of Turkish descent was simultaneously translated into Turkish (due to limited German language proficiency) by a bilingual Turkish project partner who acted as a comoderator. Participants completed a brief sociodemographic questionnaire anonymously in conjunction with the focus group. Semistructured, pilot-tested interview guides alongside a moderator guide were used to guide the discussion. Interview guides were matched on key themes and covered participants’ requirements and needs regarding a shared patient-centered, Web-based medication platform. Topics covered in the focus groups, as they pertain to this paper, included open-ended questions and probes to encourage a broad discussion about participants’ experiences with their medication management, attitudes, and opinions toward using a Web-based shared medication platform and its technological and content requirements (see Multimedia Appendix 1 for sample questions). At the beginning of each focus group, the moderator briefly presented the general idea of a medication platform by a PowerPoint presentation. Postfocus group debriefings were conducted, and central themes were documented in a research diary. Each focus group lasted between 110 and 130 min, and was audio- and videotaped. Recruitment of new participants ceased when no new themes emerged in the group discussions [37].

### Characteristics of Participants

Most patients had complex medication regimens (≥5 different types of medications taken regularly per day, not restricted to diabetes medication) and had on average 3 other chronic conditions. Of 25 patients, 16 (64%) reported having access to a personal computer/laptop and about half stated to use the internet for medication-related information. Almost all HCPs had an internet connection in their practice, and 8 out of 13 GPs (62%) reported to use electronic decision support systems. Tables 1 and 2 present demographic characteristics, computer/internet use, and recruitment of patients with T2DM and HCPs.

### Data Analysis

Audio- and video recordings were fully transcribed by trained staff with anonymity of participants completely protected and reviewed by the moderator (GB) for accuracy. The observers’ notes and debriefing notes were synthesized and integrated into the data analysis process. Data were analyzed iteratively using qualitative content analysis to structure material in codes (labels of condensed meaning units), subcategories, and categories (themes) [38-40].

**Table 1:** Characteristics of patients with type 2 diabetes who participated in the focus groups.

Patient characteristics		Patient focus groups (N=25)
Gender (female), n (%)		7 (28)
Age (years), mean (SD); range		64 (8.6); 49-77
Diabetes duration (years), mean (SD); range		13.9 (10.6); 0.8-38
Number of other chronic conditions, mean (SD); range		3.4 (1.6); 1-7
**First language, n (%)**		
	German	18 (72)
	Turkish	7 (28)
**Number of different medications taken regularly per day^a^** **, n (%)**		
	1-2 medications	2 (8)
	3-4 medications	6 (24)
	5-6 medications	5 (20)
	≥7 medications	12 (48)
**Diabetes medication, n (%)**		
	Oral hypoglycemic agents only	13 (52)
	Insulin only	3 (12)
	Oral hypoglycemic agents and insulin	9 (36)
**Education/school years, n (%)**		
	Secondary school (9 years)	12 (48)
	Secondary modern school (10 years)	5 (20)
	Grammar school (13 years)	8 (32)
Computer/laptop at home, n (%)		16 (64)
Internet use at home, n (%)		14 (52)
Internet use for medication-related information, n (%)		12 (48)
**Recruitment through, n (%)**		
	Self-help groups	15 (60)
	Heidelberg University Hospital	6 (24)
	General practitioner practices	4 (16)

^a^Not restricted to diabetes medication.

Development of thematic categories was guided by priori objectives identified in the interview guide while also allowing new themes to emerge from the data [40]. Moreover, 2 researchers (GB, CM) independently read transcripts and notes thoroughly and then coded data to establish subcategories and categories through consensus. At first, transcripts were deductively analyzed by assigning initial categories corresponding to the interview guide. Next, material pertaining to each category was analyzed inductively to refine subcategories. If the data revealed new information not fitting the preliminary coding scheme, categories were developed inductively. Throughout the iterative process of revisiting the data and connecting them with new insights, an initial coding scheme was established [40].

The researchers used Atlas.ti (Version 7.0.80, Scientific Software Development GmbH, Berlin, Germany), a qualitative software package, for organizing and coding the data. Researchers met regularly throughout the study to discuss categories and subcategories until consensus on the final set of categories was reached. By taking into account the number of focus groups reporting specific requirements, prioritization of requirements was possible.

**Table 2:** Characteristics of participating health care professionals. DMP: disease management program. N/A: not applicable. PC: personal computer.

Health care professional characteristics		Professional focus groups	
		General practitioners (N=13)	Health care assistants (N=10)
Gender, (female), n (%)		6 (46.2)	10 (100)
Age (years), mean (SD); range		54.1 (9.2); 35-64	38.6 (11.8); 21-52
**Structure of practice, n (%)**			
	Solo practice	4 (30.8)	4 (40)
	Group practice	7 (53.8)	6 (60)
	Practice sharing	1 (7.7)	
	Ambulatory health center	1 (7.7)	
**Location of practice, n (%)**			
	City center	6 (46.2)	5 (50)
	Suburbia	5 (38.5)	1 (10)
	Rural area	2 (15.4)	4 (40)
Years of work experience, mean (SD); range		24.5 (9.8); 6-40	15.5 (12.5); 0-35
Participation in DMP diabetes type 2, n (%)		13 (100)	10 (100)
Solely electronic documentation, n (%)		6 (46.2)	3 (30)
Use of electronic decision support systems, n (%)		8 (61.5)	N/A
Internet connection in practice, n (%)		12 (92.3)	9 (90)
PC with practice software connected to internet, n (%)		9 (69.2)	8 (80)
**Recruitment through, n (%)**			
	Academic teaching practices	12 (92.3)	8 (80)
	Research practices	1 (7.7)	2 (20)

## Results

### User Requirements Regarding Functionalities and Usability of a Shared Patient-Centered, Web-Based Medication Platform

Focus group participants discussed their requirements in terms of functionalities and usability of a shared patient-centered, Web-based medication platform. Categories and illustrative quotes are presented in more detail in the following section. Overall, GPs and HCAs had similar requirements regarding the medication platform as the great majority of codes were mentioned by both groups. Thus, data from GPs and HCAs were pooled together. For publication, the coding scheme and quotations were translated into English by the first author (German-native and fluent speaker of English) and thereafter cross-checked by an English- and German-native speaking coauthor (CM). Unique identifiers are used to protect participants’ anonymity (P, patient; GP, general practitioner; HCA, health care assistant; FG, focus group). To facilitate readability, categories, subcategories, and associated codes are presented in Tables 3 and 4. Moreover, requirements were prioritized (+-++++) based on the number “(1-4)” of patient and HCP focus groups reporting a specific requirement.

### Functionalities of the Medication Platform

Participants’ expectations regarding functionalities of the medication platform were divided into 5 subcategories: (1) security, access control, and supported data entry; (2) safety alerts, reminders, and notifications; (3) tracking medication history; (4) support features; and (5) electronic messaging and information sharing (see Table 3 and Multimedia Appendix 2). Although patients and HCPs had mostly similar expectations regarding functionalities, they had controversial views on automatic interaction checks and safety alerts for patients and on patients’ ability to control access to the platform.

**Table 3:** Required functionalities of the medication platform.

Subcategory and code		Patient^a^	HCP^b^
**Security, access control, and supported data entry**			
	Data security and privacy	++++	++++
	Rapid access in case of emergency	++	++
	Patient can customize and restrict access to platform	+++^c^	
	Physicians and HCPs need full access		+++^c^
	Restrict entering and changing information in platform		+++
	Simple data upload, automatic spell, and plausibility check	+++	+++
	Interoperability with management software systems		++++
**Safety alerts, reminders, and notifications**			
	Automatic interaction checks and safety alerts, trigger alert messages and visual clues to highlight interactions, risks and contraindications, what to do and specific instructions for safe use	++++^c^	
	Only high-severity drug-drug interactions, allergy alerts, contraindications, duplicate medications, and what to do (HCP perspective: physician judgment is needed)		++++^c^
	Highlight potentially hazardous medications and provide specific precautions	++	+
	Signalize new entries and changes made (eg, pop-up, colored)	++	++
	E-reminder to undertake medication reconciliation, counseling, and review of therapy		++
	E-reminders to support medication intake or discontinue intake	+	
**Tracking medication history**			
	Complete medication regimen	+++	++++
	Date of prescription, medication change, and update	++	++
	Person who entered or changed information		++
	Reason for changes or discontinuing medication	+++	++++
	Occurrence of adverse drug events	+++	++++
	Medication dispensing information from pharmacy		++
	Patients can add specific information (eg, over-the-counter medications, symptoms)		++
**Support features**			
	Search function	++	++
	Medication possession calculator	++	+
	Insulin dose calculator	+	+
	Medication plan and information can be printed, and send electronically	+++	+++
**Electronic messaging and information sharing**			
	Exchange of experiences and information between patients	+	
	Electronic messaging between HCPs		+

^a^Requirements of patients with type 2 diabetes, prioritized according to the number (1-4) of focus groups reporting requirement.

^b^Requirements of health care professionals (HCP; general practitioners and health care assistants), prioritized according to the number (1-4) of focus groups reporting requirement.

^c^Controversial views between patients and health care professionals.

**Table 4:** Requirements regarding usability of the medication platform.

Subcategory and code			Patient^a^		HCP^b^
**User interface**					
	Structured information according to diagnosis or therapeutic indication, long-term and on-demand medication	+++		++++	
	Structured information in a chronological order	++++		++++	
	Intuitive design and navigation, tailored to users’ workflow	++++		++++	
	Ergonomic presentation, large font size, customizable adaptation of information density	+++		+++	
**User-centered provision of information**					
	Lay and multilingual language, for example, evidence-based information	+++		+++	
	Glossary to support comprehensibility of medical terms, wiki to answer important questions	++		++	
	Use of visual aids, clues, and videos to facilitate understanding of information	+++		++	
	User guide, provision of training, and links to additional support	++			

^a^Requirements of patients with type 2 diabetes, prioritized according to the number (1-4) of focus groups reporting requirement.

^b^Requirements of health care professionals (HCP), prioritized according to the number (1-4) of focus groups reporting requirement.

#### Security, Access Control, and Supported Data Entry

Data security and privacy issues were intensively discussed across all focus groups, and appropriate security and access methods (eg, secure authentication) were fundamental for patients and HCPs to use the platform. Participants stressed the platform would contain sensitive information on diagnoses and medications, which was potentially valuable for third parties (eg, insurance companies, pharmaceutical industry). Hence, several patients and HCPs mentioned concerns to become “gläsern” (transparent, P2-FG3) and monitored by the platform:

I would not use it if it would be cumbersome to use. That would be the first requirement—the handling. And I would not use it if it had negative effects for the patients or if I would expect more control of my work, when I feel that it is going to be a surveillance tool for my work or when I witness that insurances, border authorities or someone else is interested in these data.

GP2-FG4

Accordingly, participants stated they had to decide whether the benefits of using the platform would outweigh the theoretical risks. On the other hand, participants requested an emergency access functionality in the platform to enable physicians’ rapid access to a patients’ current medication list and important patient-related information (eg, allergies, intolerances, risk factors). To be acceptable to them, most patients emphasized they need to be in control of their data and the authorization of different HCPs and significant others to access and add information to their personal account in the platform:

The access would have to be very restricted and controlled by myself.

P2-FG1

Although most participants supported the general idea of a patient-controlled platform, most HCPs did not support patients’ ability to withhold information or restrict access to certain information. HCPs stressed that physicians need to be fully informed about a patient’s regimen to make informed decisions. Furthermore, participants had extensive discussions about who should be able to enter and change information in the platform.

Most HCPs thought it was important to restrict entering and changing information in the platform. For instance, physicians can enter their own prescription but should not be able to change medications prescribed and entered by other HCPs.

Furthermore, HCPs suggested that patients can enter over-the-counter medications (OTCs) and symptoms experienced in the platform but should not be allowed to change or delete a physician’s prescribed medication. Otherwise, HCPs stressed “I can no longer trust my own case” [GP1-FG4] and perceived this would affect their liability as well as the reliability of information in the platform. Likewise, one HCA emphasized:

The patient should have the possibility to add something [to the platform], but it should be clear that it comes from the patient, yes, so one always knows, he [the patient] has added something. But he [the patient] should not be able to delete anything…

HCA1-FG2

Besides, HCPs mentioned concerns about the accuracy of patient-entered data. Although some patients stated they wanted to enter and update information themselves, patients with limited computer experience or skills said they wanted their GP, functioning as a coordinator, to enter or upload prescription information. Others stated they would ask relatives or friends to support them entering information and using the platform.

A fundamental requirement for patients and HCPs was that the platform facilitated easy access, entry, and upload of medication information while maintaining high security standards. For instance, both groups perceived an automated entry of medication information was important for ease of use and a prerequisite for acceptance of the platform.

The HCP groups underlined that patients with T2DM often get prescriptions from different providers, for instance, GP, specialist, or hospital, and buy OTCs directly at the pharmacy. Thus, the platform should interoperate with different management software systems and enable an automatic data upload. Many HCPs stressed they would not enter medication information twice, in their own system and in the medication platform:

The upload from the practice management software should be automatic, that it isn’t more work to open the platform in the practice management software. I think this is very important because when there is an administrative effort, it becomes difficult, but if there are interfaces to the practice management software, then it’s certainly a good thing…

GP1-FG4

Some patients suggested adding new medications to the platform could be simplified by scanning the medication barcode with their mobile device or a barcode scanner. To illustrate this suggestion, 1 patient said:

We have also heard here that we are sometimes simply overwhelmed with our medications… because they either constantly change or something else. Thus, the system should be designed in a way that it is relatively simple for me to put in my medications. And I have to keep it [medication] up to date. I mean if this is too complicated or takes too much effort… it is not up to date. Then it’s of no use… as I said, if I handle the system self-responsible, it must be easy, a medication package has a barcode. Quickly, with the mobile phone the number is scanned. Quickly, the medication is entered. If I have to enter everything each time by hand… that’s just too much.

P5- FG4

Others emphasized if they entered a medication manually, the platform should feature an automatic spell and plausibility checker and provide an automatic word completion for a quick entry.

#### Safety Alerts, Reminders, and Notifications

Safety alerts, reminders, and automated notifications were well-discussed in all focus groups with differences detected in patients’ and HCPs’ expectations. Most patients perceived they lacked information on potential side effects, long-term effects, and drug interactions of their prescribed medication and voiced concerns regarding the safety of their regimen. Thus, many patients reported to seek risk-related information from a range of sources including their GP, specialist, community pharmacist, local self-help group, friends, as well as Web-based (internet) resources.

Accordingly, patients suggested the platform should provide comprehensive risk-related information and automatically check and highlight interactions (eg, drug-drug, allergy, food) in their regimen.

I have to take lots of different medications…there [patient information leaflet] you can read about incompatibilities with this and that substance, but I don’t even know at all which substance is in which medication…it would be important, if I have my medications in such a system that it automatically reconciles: “Do these fit together at all?” I mean, I always have to trust my doctor that he knows this, but sometimes I have the feeling: “How does he know all of this?” Because there is so much stuff he has to know. Sometimes I have a bad feeling, whether he really knows that…I have an insecurity with the medications…I often have the feeling it is a calculated risk…

P5-FG4

Thus, several patients wished real-time safety alerts to pop-up automatically indicating the severity of interactions in their regimen, for instance, by using distinctive color-coding (eg, according to traffic light, red=serious). On the other hand, 2 patients were also averse to receiving information on potential adverse effects, as this may negatively influence their attitude toward a medication.

Likewise, HCPs expressed hesitation about offering an automatic interaction check to patients and mentioned concerns about how patients dealt with this critical information, as in their view, this could increase patient fear, encouraging nonadherence, and numerous discussions. HCPs also noted that interactions in the regimen of chronically ill patients are common, often not avoidable, and needed to be judged by them. Over half of the GPs reported to use electronic decision support systems for prescription writing. Although GPs generally valued drug interaction and allergy alerts during prescribing, they stated to override these frequently due to little clinical significance and extensive numbers of warnings. Nonetheless, HCPs believed that patients need to be informed about the most relevant adverse and long-term effects to monitor their own treatment and know how to reduce potential risks. Thus, HCPs suggested only showing relevant adverse effects, contraindications, duplicate medications, allergy alerts, and high severity drug-drug interactions to patients. At the same time, HCPs, however, also recognized difficulties regarding liability and legal implications when only certain warnings would be displayed in the platform. The following exchange exemplifies this:

There are warnings; you have to go deaf, this doesn’t help…only the absolute relevant warnings should pop-up…

GP1-FG1

Response of another participant:

This is not possible, because it is actuarially all relevant, that’s the problem.

GP3-FG1

When with every ACE inhibitor and potassium-sparing diuretic it pops-up every time… we know that, and this doesn’t help. So, it should really only be what is relevant… so that the absolute no-go’s pop-up.

GP1-FG1

Both groups pointed out that it was necessary to concisely describe the actions to be taken by patients in lay language in the alert to mitigate potential adverse effects and to promptly contact their treating physician.

A few participants also requested that the platform notified users about potentially hazardous medications (eg, anticoagulation) or medications with unclear benefits. In addition, patients and HCPs thought it was necessary to highlight new entries or changes made in the platform automatically, for instance, by using visual clues (eg, pop-up, color):

That you do not have to check constantly if something has been changed, at what time something has been changed, but that this actually runs automatically…

HCA3-FG2

In general, HCPs saw great value of the platform for medication reconciliation and counseling by combining all relevant medication information of a patient in one place. Hence, HCPs were interested in setting up an automated prompt that reminded them periodically to undertake medication reconciliation and counseling. Similarly, some patients wished the platform enabled setting up tailored audible medication-taking prompts that reminded them to take their medications at specific time intervals or, for instance, to discontinue intake before surgery. To enable this feature, patients suggested the platform should be linked to their mobile device.

#### Tracking Medication History

Tracking a patient’s medication history received great attention in the group discussions and was perceived by all participants, especially HCPs, as an essential component of the platform. Participants were enthusiastic about the platforms’ potential to increase the ease of documentation and produce a structured presentation of a patient’s complete medication history (eg, including prescription and nonprescription medication, supplements), thus facilitating information exchange and medication reconciliation across health care sectors and professionals. HCPs underscored current challenges (eg, resource and time constraints, lack of cross-sectorial collaboration, and technical interoperability, drug discount contracts) in reconciling medications of their chronically ill patients, particularly during transitions of care. For the patient groups, capturing their medication history was relevant to see how their condition and treatment developed over time. Several patients described how they kept track of their currently and previously used medications by creating paper-based medication lists where they documented changes (eg, regarding dosage, frequency, and generic substitution) made in their regimen and related these to clinical parameters (eg, glycated hemoglobin):

What is important for me, I always make a note when I changed the dosage or the medication […] that I can enter it [in the platform] and that I know the dosage has been […] that’s a thing I like to check. Has it improved or worsened since the change. I found this was very important and have written it down next to it.

P3-FG1

Moreover, patients stressed that medication changes are common, often tied to specific problems or therapeutic goals, and without documentation, they would have difficulties keeping track of this information. HCPs underlined often not being fully informed about a patient’s medication regimen as only the patients are in the position to account for their self-medication. Thus, patients should enter OTCs and symptoms experienced to complete medication history taking. HCPs, however, experienced that patients had difficulties disclosing their co-usage of OTCs, vitamins, or herbal supplements. Hence, HCPs suggested patients would need prior guidance on the importance of accurately documenting prescription and nonprescription medications in the platform. Above all, participants across focus groups emphasized it was crucial to consistently record reasons for changing or discontinuing medications in a patient’s medication history, including information on side effects, ADEs, and intolerances. This information was perceived vitally important to make informed treatment decisions and ensure patient safety. In addition, participants stressed the platform should automatically capture the name of the person (eg, patient, prescribing physician, dispensing pharmacy) entering data as well as the date of each transaction:

I would like to have a medication history, who prescribed and discontinued what, when and why…because often with chronically ill patients there are let’s say circular procedures: Medication A, Medication B, Medication C, Medication A and then the question arises: did he not tolerate it? After some time, the patient doesn’t know it anymore and I have to admit sometimes I’m not either…to have a comment field to record the reason for discontinuing the medication, due to intolerance, allergy, medication change, hospital stay, ineffectiveness or so on.

GP2-FG4

Documentation of possible adverse drug reactions with date of occurrence. That there is somewhere a note, there have been adverse effects. After five years I forgot that one [a patient] on Amlodipin got edemas and I will prescribe it again, then it happens again. There are also more severe adverse effects…

GP2-FG2

If a medication is changed that the date is recorded when it was changed and what was prescribed instead…

HCA2-FG2

Furthermore, in participants’ view, medication dispensing information from the pharmacy (eg, date of dispensing, generic substitution, and initials of dispensing pharmacy) would shed light on generic substitution and may improve patients’ comprehension. Thus, the platform should clearly link the patients’ prescribed medication with the dispensed generic medication.

#### Support Features

Participants suggested incorporating support features to facilitate retrieval of information in the platform and to help organize daily medication taking. Due to large amounts of data stored in the platform, participants stressed the platform should contain a search function allowing rapid retrieval of information. To support patients’ self-management, many participants wished to be able to print relevant information (eg, medication list, specific directions for use, and administration) as well as to send information electronically. Participants also highlighted they would benefit if the platform offered a feature to calculate medication possession (ie, number of refills remaining) and the amount of insulin units needed to reach a target blood glucose concentration, as the following discussion illustrates:

It would be practical…if the platform would list, when one has to get a refill, so one can plan ahead…

HCA1-FG3

I have another idea. I have insulin-dependent diabetes and fly to New York…or further [across several time zones] How do I have to adjust my insulin dosage now? There are great calculations models available…

GP3-FG3

So, I would even say that one could also specify the time period, so that one knows how much he has to take with him…

HCA1-FG3

#### Electronic Messaging and Information Sharing

The potential of the platform to enable electronic messaging among users was only discussed briefly and revealed controversial aspects among focus group participants. A small number of patients said they would like to share information and personal experiences, for example, with certain medications, adverse effects, or alternative treatment options, with their peers through an integrated anonymous chat or online user forum:

I have recognized it right from the beginning how each of us told about his illnesses and so on. I heard about this and that. Aha! That’s the same with me. There [relating to the platform] it is possible to exchange certain experiences. Surely, I cannot say, […] you have to do this and that. That doesn’t work. But you can get suggestions and maybe you see: others are in the same situation. He has psychological problems with all of that—same with me. I know I am not alone. There are others who have the same problems. This experience by itself is very helpful…

P5-FG4

Although patients generally saw value in exchanging experiences with their peers, they also voiced concerns regarding reliability and credibility of information exchanged. A few HCPs responded positively to communicate with other HCPs regarding a patient’s medication regime (ie, to resolve discrepancies) through the platform, particularly during transitions of care. However, the majority of HCPs were reluctant to communicate electronically with other providers and, especially not with patients, and feared an increased workload. Furthermore, they emphasized it should not be used for urgent matters as it would be impossible to answer requests in a timely manner:

...I imagine regular consultation hours. I don’t read emails or do chats. I have no time for that during consultation hours; I must say clearly. In our practice, we have even stopped interruptions by telephone except in real emergencies...

GP4-FG3

### Usability of the Medication Platform

Participants’ expectations regarding usability of the medication platform were grouped into 2 subcategories: (1) user interface and (2) user-centered provision of information (see Table 4 and Multimedia Appendix 3). Overall, patients and HCPs expressed similar expectations on the issue of usability.

#### User Interface

Patient and HCP groups emphasized that medication information should be displayed in a clear and logical manner in a large font size on the platform. For instance, participants suggested listing medications in a chronological order, organizing them according to diagnosis (eg, T2DM, cardiovascular), and categorizing medications in relation to their short-term (eg, an antibiotic) or long-term use (eg, OHA):

The medication platform should be simply structured, there should be the long-term medications, then the on-demand medications or short-term medications, and easily accessible and changeable…

GP3-FG1

Overall, to support a patient’s self-management, participants highlighted that the platform should be easy to navigate, to enable quick access to relevant medication information, and to observe a patient’s current medication at a glance. After log in, the current medication list should be prominently displayed on the front page, thus enabling a quick overview of a patient’s current medication. Likewise, participants commented it was important that the platform provided sufficient information to make informed decisions but at the same time was not overloaded with information:

However, one should not overload such a system, because otherwise you don’t find anything and have to search…

GP4-FG3

Consequently, participants requested the platform should enable users to customize information density. For instance, some participants suggested to provide access to detailed information (eg, regarding side effects) via a link on the specific medication. Especially HCPs groups expected the platform to be tailored to suit their practice workflow and to function quickly and reliably.

#### User-Centered Provision of Information

From the participants’ point of view, a user-centered provision of information in the platform was fundamental for the adoption and successful use by patients and HCPs. Most patients described their difficulties understanding medication information in consultations and in written information leaflets. Accordingly, patients as well as HCPs stressed that information should be evidence-based and provided in “nontechnical jargon” [P6-FG4] in the platform. In addition, the group of Turkish patients remarked it was important that the platform provided access to multilingual information. Both groups thought it would be beneficial to provide a glossary or encyclopedia to support comprehensibility of medical terms and a wiki for answering important and frequently asked questions:

Yes, common misunderstandings, frequently asked questions and common medication intake errors…

HCA4-FG2

Especially the patient focus groups highlighted they would benefit if visual aids (eg, icons, pictograms, images of medications, daily injection plan) and videos (eg, instruction video) were included in the platform to assist identifying, understanding, and using their medications appropriately:

When it comes to injection technique, a video would be very helpful…

P2-FG1

HCPs also emphasized to integrate visualization methods into the platform:

It would be great if you click on a medication a video is shown or something else…

GP2-FG2

Besides, patients’ desired prior instruction and training for using the new system and some requested a toll free hotline and a user guide to aid navigation. Furthermore, patients wished the platform linked them to an expert helpline for personal medication counseling and further self-management support (eg, regional patient support groups).

## Discussion

### Principal Findings

The qualitative UCD approach enabled a deeper understanding of the requirements of patients with T2DM, GPs, and HCAs regarding functionalities and usability of a shared patient-centered, Web-based medication platform. Identifying key users’ requirements early on is a critical step in the development and implementation of a new system to successfully support medication management and treatment of patients with T2DM and their HCPs.

In patients’ view, a medication platform offers potential to improve their understanding, address their medication-related concerns, and support their medication self-management activities. HCPs, in turn, focused on the platforms’ ability to aid comprehensive medication history taking and reconciliation across health care settings. Appropriate security and access methods, supported data entry, printing and sending information electronically, and tracking medication history were perceived by participants as essential functionalities. Although patients wanted automatic interaction checks and safety alerts, HCPs, on the contrary, were concerned that unspecific alerts confuse patients and lead to nonadherence. Furthermore, HCPs were opposed to patients’ ability to withhold or restrict access to information in the platform. To optimize usability, there was consensus among participants to display information in a structured, chronological format, to provide information in lay language, to use visual aids and customize information content, and align the platform to users’ workflow.

Most participating patients had safety concerns, and prior research suggested that patients most commonly avoid taking their medications due to concerns about adverse effects [5]. To address these concerns, patients desired comprehensive risk-related information, automatic interaction checks, and safety alerts in the platform. Undeniably, patients play a central role in managing medication-related risks, and need to be engaged in self-monitoring to improve medication use [41]. Keeping in mind that patients with T2DM often have complex medication regimens and receive pharmaceutical treatment from different providers, tailored and clinically meaningful safety alerts combined with clear instructions on how to proceed could facilitate early detection and reduce serious complications. On the other hand, some patients may not want or feel capable to use safety-related IT apps. Thus, it will be necessary to customize safety alerts to the specific needs of individual patients, for instance, to allow triggering the interaction check manually (ie, non-interruptive) or showing only high severity alerts. To date, however, lack of specificity and low sensitivity of medication alerts in clinical decision support systems is still a problem [42,43]. Comparable with HCPs’ concerns, unmodified medication safety alerts without concurrent physician interpretation may create confusion and anxiety among patients and thus impede medication-taking. At this point, it is important to emphasize that the medication platform does not replace or substitute for patient-provider consultation but has the potential to complement instructions and self-management support given. Furthermore, the platform offers potential to transmit essential information (eg, access to complete medication regimen, diagnoses) among members of the health care team. This may, for instance, promote stronger physician-pharmacist collaboration to improve medication therapy and safety. Overall, however, HCPs had conflicting views regarding the provision of risk-related information in the platform. Most HCPs held negative attitudes toward safety alerts for patients and also anticipated an increase in their workload. Nevertheless, they perceived patients should be made aware of the most relevant adverse effects. Delbanco and colleagues [44] found that patients who had electronic access to care providers’ notes felt more in control of their care, and reported improved medication adherence and minimal concerns without increasing providers’ workload. Hence, unintended consequences to patients need to be explored further in the user-driven design process to see if the platform and specifically safety alerts are both beneficial and acceptable to patients and HCPs and exceed potential risks [43]. Perhaps patients may have a more sensible approach to safety alerts than HCPs fear.

Patients’ ability to control and restrict access to medication-related information or change physicians prescribed medication evoked great concerns (ie, medicolegal liability) among HCPs and was perceived to threaten physicians control and the quality of care. Thus, patients’ desired ownership over the platform seems to clash with the predominant approach held by HCPs. Similar to previous research [45], physicians were concerned to make suboptimal decisions about a patient’s treatment due to incomplete or inaccurate information. In contrast, Haverhals et al [26] also concluded that health apps should provide patients the ability to selectively disclose information (eg, alternative medications) to different HCPs. Further research and ongoing involvement of intended users is therefore necessary to elaborate how to place control of the medication platform in the hands of patients while accounting for HCPs’ needs. Moreover, introducing such a patient-held information system challenges current structures and requires a shift in patients’ and HCPs’ roles and responsibilities.

One of the greatest concerns among patients and HCPs were privacy and security issues, which seems more prominent in Germany than in many other countries, and thus delay a national e-medication initiative [31]. Security and privacy concerns, however, have also been identified previously as a potentially large barrier to personal health record use [16,46]. Further investigation of privacy, security, and legal concerns is needed to better understand what prompts these concerns by patients and HCPs and to ensure that users’ concerns are adequately addressed. Moreover, ways of granting secure and effective emergency access to the medication platform need to be explored in the iterative development process.

Participants were enthusiastic about the platforms’ potential to collect and store a patient’s complete medication history in a structured format by engaging both patients and HCPs. Indeed, medication errors are common in primary care and there is a need for better monitoring, patient education, and improved communication between patients and their HCPs [8,47]. Although participants positively viewed patients’ ability to contribute their self-medication and symptoms (eg, ADEs, intolerances) to the platform, some HCPs also mentioned concerns regarding reliability and accurateness of patient-entered data. Tang and colleagues [16] emphasized that the reliability of data entered by patients depends on the nature of information per se, the patient’s literacy level, and the motivations for recording the data. Providing data entry functionalities in the platform, for example, scanning the medication barcodes with a mobile device and integrating a plausibility checker, may help to improve information accuracy. Moreover, effective training of patients and HCPs will be a prerequisite to unfold the platform’s full potential. Overall, facilitating patient engagement in medication history collection seems a promising approach to improve medication reconciliation, patient-provider communication, and thus patient safety [48].

Although the platform may offer new ways to mediate communication among patients and HCPs, this function received only little attention in the focus group discussions and was subject to concerns. Although patient medication reviews have also been identified previously as a valuable complementary source of information for patients [49], the reliability of this information has likewise been questioned [50]. Nevertheless, patient online communities have been suggested to facilitate patients’ comprehension, informed decision making, and medication self-management [51]. Despite evidence that patients increasingly desire Web-based patient-provider communication [52], patients in this study did not request Web-based messaging with their HCPs. Maybe patients did not expect it to be an effective way to communicate with their HCPs about their medications or generally lacked ideas how the platform could facilitate patient-provider communication. Likewise, HCPs were reluctant to communicate electronically with patients, although a few HCPs saw potential for medication reconciliation through secure messaging with other HCPs.

Patients and HCPs had similar expectations regarding usability of the medication platform. For both groups, it was essential that the platform structured information chronologically in an intuitive, user-centered format (eg, customizable content in lay and multilingual language) on 1 screen and aligns to users’ workflow. As also proposed earlier [16,30], special attention needs to be paid to health literacy issues when developing such a platform. Visual aids, pictograms, and customized videos requested by participants can enhance patients’ understanding of how they should take their medications [53,54]. However, there are additional skills in terms of accessing and effectively using HIT, subsumed as eHealth literacy, that are required to fully engage with eHealth resources [55]. To date, patients and HCPs have not been trained sufficiently in the optimal use and implementation of HIT in medication management [56]. It is therefore vital that all essential aspects of using and implementing the medication platform should be included in the training, telephone, and on-site support [57]. Moreover, prior education of patients and HCPs on medication self-management (support) is vital to equip users with the essential skills and thus have implications for realizing the potential benefits of the platform. Further developments of the platform should also explore and incorporate features (eg, electronic diary, self-monitoring tools, nutrition module) that help patients to adopt and maintain a healthy lifestyle [2].

### Strengths and Limitations

A strength of this qualitative study is that the in-depth perspectives of patients with T2DM who were diverse in age, duration of diabetes, and on a variety of medication regimes and their primary HCPs, including GPs and HCAs, were collected. By applying the principles of UCD, intended users have been involved early in the design and development process of the medication platform and thus increase the likelihood to achieve a functional and useful system. Although participants were purposefully selected and recruited from different health care settings, they may not be generalizable to the diabetes patient population or HCPs overall. As participants “opted in” to the focus groups, they may have greater interest in medication management and HIT, and may represent the perspectives of “early adopters,” although computer experience differed among participants. Although this provides valuable insights on the needs of early adopters of HIT, we do not know the perspectives of potential participants who chose not to participate. Moreover, this study did not specifically focus on low-literate patients, their caregivers, or other HCP groups. Incorporating their perspectives may have generated a more extensive requirements elicitation. Despite the limitations, this study enables a comprehensive description of patients’ and primary HCPs’ requirements regarding a shared patient-centered, Web-based medication platform and will ultimately help to design the platform according to these needs.

### Conclusions

The need to explore new approaches to facilitate medication management and treatment across health care sectors is an important issue that becomes increasingly important with the number of patients with T2DM. Given that patients are in control of their daily diabetes care [58] and the central users of the prospective system, their requirements need to be taken into account. This must, however, always be regarded in relation to the respective health literacy of each patient. No “one-size-fits-all” solution seems to be possible. The platform will be needed to be tailored to patients’ needs and capabilities. Furthermore, reconciling differences in requirements of patients and HCPs, especially regarding medication safety alerts and access control, will necessitate substantial engagement by all intended user groups in the ongoing development process. Balancing patients and HCP’s preferences is a prerequisite to empower patients and improve medication management and safety, while encouraging HCPs to use the platform. Once the prototype is developed, its evaluation will show how patients and HCPs evaluate and use the system, showing if the system has a good FITT and promotes the intended health outcomes.

## Appendix

Multimedia Appendix 1 Sample questions used in the focus groups with patients and healthcare professionals.

Multimedia Appendix 2 Overview of required functionalities of the medication platform.

Multimedia Appendix 3 Overview of requirements regarding usability of the medication platform.

## Abbreviations

ADE: adverse drug event
DMP: disease management program
FITT: Fit between Individuals, Task, and Technology
GP: general practitioner
HCA: health care assistant
HCP: health care professional
HIT: health information technology
Infopat Rhein-Neckar: Information Technologies for Patient-Centered Health Care
OHA: oral hypoglycemic agents
OTC: over-the-counter medications
PC: personal computer
T2DM: type 2 diabetes mellitus
UCD: user-centered design
