# Supplementary material for: Developing a Shared Patient-Centered, Web-Based Medication Platform for Type 2 Diabetes Patients and Their Health Care Providers: Qualitative Study on User Requirements
Source: J Med Internet Res. 2018 Mar 27;20(3):e105. doi: 10.2196/jmir.8666 (PMC5893891; doi:10.2196/jmir.8666)
Supplement: Multimedia Appendix 1 [file jmir_v20i3e105_app1.pdf]

Textbox 1. Sample questions for patient focus groups.

General Introduction

- Introduction of the moderator, co-moderator and note taker; background information about the study and purpose of the focus group discussion; protection of privacy; introduction of participants

Part 1: patients' experiences with their medication management

- What information did you receive about your medication regimen/your medications?
    - Do you feel sufficiently informed about your medication regimen/the medications you are taking? Please give an example.
- Who or what could support you in the self-management of your medication regimen/your medications? Please express your expectations.

Part 2: patients' attitudes and requirements regarding a shared web-based medication platform

Presentation of the general idea of a shared patient-centered web-based medication platform.

- What comes to your mind when you hear about this concept?
- Imagine that you could design the perfect medication platform. What information about your medication should be displayed on the platform?
    - What information do you think is essential and needs to be available?
- How should this information be presented and retrievable from the platform to support you in self-managing your medications? Please express your expectations.
    - What functionalities do you think need to be included in the platform?
- Who (e.g. patient, health care professional, others) should be able to view and/or access certain information on the platform?
- What advantages or benefits could this web-based medication platform have for you (e.g. for your medication self-management, for consultations with health care professionals and others, when visiting different physicians)?
- What do you think are potential drawbacks of such a web-based medication platform?
    - What are reasons for you not to use the platform?

Closing

- Summary of requirements elicited in the focus group discussion.
- Is there anything else that is important to you that has not been addressed yet?

Textbox 2. Sample questions for focus groups with health care professionals.

General Introduction

- Introduction of the moderator, co-moderator and note taker; background information about the study and purpose of the focus group discussion; protection of privacy; introduction of participants

Part 1: professionals' experiences with their medication management practice

- What information do you provide for type 2 diabetes patients about their medication regimen/their medications?
    - How do you provide this information to your patients? Please give an example.
- How should medication counseling be ideally organized and carried out in general practice to support patients' self-management?
    - How could you be supported to perform medication counseling with type 2 diabetes patients?

Part 2: professionals' attitudes and requirements regarding a shared web-based medication platform

Presentation of the general idea of a shared web-based medication platform.

- What comes to your mind when you hear about this concept?
- Imagine that you could design the perfect medication platform. What information about a patients' medication should be displayed on the platform?
    - What information do you think is essential and needs to be available (e.g. for patients' self-management, for medication counseling)?
- How should this information be presented and retrievable from the platform (e.g. to support patients' self-management, your medication counseling)? Please express your expectations.
    - What functionalities do you think need to be included in the platform?
- Who (e.g. patient, health care professional, others) should be able to view and/or access certain information on the platform?
- What advantages or benefits could this web-based medication platform have for you (e.g. for self-management support, medication counseling and reconciliation with different providers)?
- What do you think are potential drawbacks of such a web-based medication platform?
    - What are reasons for you not to use the platform?

Closing

- Summary of requirements elicited in the focus group discussion.
- Is there anything else that is important to you that has not been addressed yet?
