# Supplementary material for: Developing a Shared Patient-Centered, Web-Based Medication Platform for Type 2 Diabetes Patients and Their Health Care Providers: Qualitative Study on User Requirements
Source: J Med Internet Res. 2018 Mar 27;20(3):e105. doi: 10.2196/jmir.8666 (PMC5893891; doi:10.2196/jmir.8666)
Supplement: Multimedia Appendix 2 [file jmir_v20i3e105_app2.pdf]

Required functionalities of the medication platform

Security, access control, and supported data entry

- **Data security and privacy**
- **Simple data upload, automatic spell check**
- Emergency access
- Patients can customize and restrict access
- HCPs[a] need full access
- Restrict entering and changing information
- Interoperability

Safety alerts, reminders, and notifications

- **Automatic interaction checks and safety alerts**
- **High-severity interactions only**
- Highlight hazardous medications
- Signalize new entries and changes
- E-reminder to support intake
- E-reminder for medication reconciliation

Tracking medication history

- **Complete medication regimen**
- **Reason for changes**
- **Occurrence of adverse drug events**
- Date of prescription, medication change or update
- Documentation who entered or changed information
- Medication dispensing information from pharmacy
- Patients can add specific information

Support features

- **Medication plan can be printed and send electronically**
- Search function
- Medication possession calculator
- Insulin dose calculator

Electronic messaging and information sharing

- Exchange of experiences and information between patients
- Electronic messaging between HCPs

Legend:
green: requirements stated by patients with type 2 diabetes only
blue: requirements stated by health care professionals only
underlined: controversial views between patients and health care professionals
bold font: relates to the requirements reported with high frequency
[a]Health care professionals
