# Supplementary material for: Developing a Shared Patient-Centered, Web-Based Medication Platform for Type 2 Diabetes Patients and Their Health Care Providers: Qualitative Study on User Requirements
Source: J Med Internet Res. 2018 Mar 27;20(3):e105. doi: 10.2196/jmir.8666 (PMC5893891; doi:10.2196/jmir.8666)
Supplement: Multimedia Appendix 3 [file jmir_v20i3e105_app3.pdf]

Requirements regarding usability of the medication platform

User interface

- **Structured information according to diagnosis, long-term and on-demand medication**
- **Structured information in a chronological order**
- **Intuitive design and navigation, tailored to users' workflow**
- **Ergonomic presentation, large font size, customizable adaptation of information density**

User-centered provision of information

- **Lay and multilingual language**
- **Glossary and wiki to support comprehensibility**
- **Use of visual aids, clues and videos**
- User guide, provision of training and links to additional support

Legend: green: requirements stated by patients with type 2 diabetes only
bold font: relates to requirements reported with high frequency
